# Teledentistry awareness among dental professionals in Saudi Arabia

**DOI:** 10.1371/journal.pone.0240825

**Published:** 2020-10-15

**Authors:** Khalifa S. Al-Khalifa, Rasha AlSheikh

**Affiliations:** 1 Preventive Dental Sciences Department, College of Dentistry, Imam Abdulrahman Bin Faisal University, Dammam, Saudi Arabia; 2 Restorative Dental Sciences Department, College of Dentistry, Imam Abdulrahman Bin Faisal University, Dammam, Saudi Arabia; Centre Hospitalier Regional Universitaire de Tours, FRANCE

## Abstract

**Background:**

Teledentistry is believed to improve dentalcare provided to patients and time management efficiency. In addition, teledentistry can be a useful tool for peer education, consultations and ensures proper channeling for referrals.

**Objective:**

This study aimed to explore Saudi Arabian dental professionals' perceptions of teledentistry's benefits in improving dental practice and patient care.

**Methods:**

A descriptive cross-sectional study involving an electronic survey of a sample of Saudi Arabian dental professionals. A validated 26-item, 5-point Likert-scale questionnaire was used to assess perceptions of dental professionals in four domains: usefulness of teledentistry for patients, the usefulness of teledentistry for dental practice; the potential of teledentistry to improve practice; and existing concerns about the use of teledentistry. Statistical analyses involved descriptive statistics, bivariate analysis using SPSS software. Significant differences were considered at a *p*-value of 0.05.

**Results:**

With an overall response rate of 28.6%, 286 dental professionals participated in the study. More than 70% of respondents agreed or strongly agreed that teledentistry would improve dental practice through enhancing communication with peers, guidance and referral of new patients. A substantial proportion of respondents (60–70%) expressed uncertainty with technical reliability, privacy and diagnostic accuracy. The participants' qualification was statistically significant with usefulness of teledentistry for patients (p = 0.027), while work experience in years was statistically significant with both usefulness of teledentistry for dental practice and patients (p = 0.046 and <0.0001 respectively).

**Conclusion:**

Generally, the feedback gained by this study showed readiness of the dental professionals to be engaged in the teledentistry approach. Further investigation of the business model of teledentistry is needed to understand the readiness and challenges. Directed campaign to educate dentists and the public of the technology and it is potential is necessary.

## Introduction

Tele-technology has received more attention in the last decade in both medical and dental fields with the potential of an easy, fast, and safe way to deliver and share health information. [1–3] Teledentistry is believed to improve dental care provided to patients as well as time management. It facilitates better referral systems based on patient's needs, decision-making, reduced waiting lists, and consultation time. [4–7] Teledentistry can extend dentalcare to patients in rural areas at a reasonable cost and convenience, reducing the need for traveling and ensures proper channeling for referrals. [8] In addition, teledentistry can be a useful tool for peer education and consultations where dentists can share their experiences and run discussions and continuous education sessions online through webinars and other online channels.

Despite the extensive use of telemedical applications in healthcare, many dentists are unfortunately ignorant of the nature of teledentistry, the benefits behind its use, or its adaption/application in routine practice. [9] Several studies focused on the perception of healthcare providers towards teletechnology in the provision of healthcare to patients. A study by Palmer et al. has investigated the awareness of orthodontists on the use of digital and electronic technology, where close to 70% of the respondents agreed with the use of teletechnology and only as few as 36% reported worrying about security and privacy issues. [10] In another study by Wood et al., they explored the demands for the use of teledentistry by general dentists and oral and maxillofacial surgeons where they concluded that teledentistry could be an essential step to address issues regarding patient access to care or healthcare cost. [11] Estai et al. assessed the perception of Australian dentists on the use of teledentistry. They stated that most dentists (80%) reported positivity regarding teledentistry and the benefits gained from implementing the technology. [12] In another study, Mandall et al. stated that up to 71% of orthodontists agreed that teledentistry would be highly beneficial for patients' referrals and half of the participants felt it would reduce the treatment time needed. [13] The perception from the patient's aspect was explored by Donelan et al., who found that more than 60% of patients reported no difference in the overall quality of the visit between virtual and conventional face-to-face. They also stated that patient convenience was enhanced by using telemedicine for follow-up and care. [14]

In Saudi Arabia, more studies have been published regarding telemedicine applications in the medical field than in dentistry. AlShaya et al. investigated the use of mobile-phones for patient diagnosis and treatment planning among children, where they found that teledentistry could be a reliable tool for the initial diagnosis of caries. [7] AlKlayb et al. developed a mobile-phone application to investigate the effectiveness of teledentistry to educate mothers. In their study, the application significantly improved mothers' knowledge of their children’s’ oral health needs. [9] Albarrak et al. assessed physicians' knowledge, perception, and attitude toward the implementation of telemedicine in clinical practice. They stated that the majority of physicians have low knowledge of telemedicine and its benefits. There were barriers mentioned by the participating physicians, mostly privacy, cost, technology, as well as lack of training. [15]

There is a limited amount of literature addressing the attitudes and awareness of dental professionals towards teledentistry and its use in healthcare provision. Therefore, this survey was aimed to assess Saudi Arabian dental professionals' perceptions towards teledentistry and its usefulness for the dental practice.

## Materials and methods

### Questionnaire instrument

An unidentified electronic questionnaire of Saudi Arabian dental professionals was carried out between March and April of 2020. The questionnaire was adapted from a similar study by Estai et al. That study was initially developed to evaluate Australian dental practitioners' perceptions of teledentistry's usefulness and its role in improving dental practice and patient outcomes. [12, 16] The first part of the questionnaire covered professional and demographic information as well as communication methods preference. The second part of the questionnaire was based on five-point Likert-type questions consisting of 26 questions, which were divided into four categories: data security concerns by the dental professionals, teledentistry and practice improvement, the usefulness of teledentistry for dental practice as well as its usefulness for dental patients. The questionnaire was pretested to a group of 20 general dentists before distribution to obtain feedback, and overall acceptability of the questionnaire and minimal corrections were made based on their response. Since this was a questionnaire-based study, an exemption was granted for this study by the Ethical Committee of the College of Dentistry, Imam Abdulrahman Bin Faisal University.

### Questionnaire distribution

This was a cross-sectional survey-based study, where an e-mail list of 1000 randomly chosen dental professionals was obtained from the Saudi Commission for Health Specialties database. The database contains information of more than 10,000 dental professional in Saudi Arabia. The random sample selected shared common professional and demographic features with the Saudi Arabian dental workforce. Furthermore, the sample was stratified by qualification and gender in order to get a more representative sample of the dental professionals in Saudi Arabia. The survey was then distributed by e-mail. There was a brief description of the questionnaire's purpose with a definition of teledentistry and its benefits and possible uses in daily practice. This was followed by a consent agreement to participate in the survey. As means for follow up with the dental professionals, a reminder e-mail was scheduled to be sent at a selected periodic interval (once a week) to all non-respondent dental professionals.

### Data analysis

The data were entered in MS Excel (2010) and transferred to IBM SPSS Statistics for Windows, version 22 (IBM Corp., Armonk, NY, USA) for statistical analysis. Descriptive statistics included means, standard deviations, and frequency distributions. A probability weighted ANOVA test was performed to evaluate differences in the mean score of responses for variables such as age, gender, work experience, and qualification. A p-value of 0.05 was used for statistical significance.

## Results

Between March and April of 2020, 1000 surveys were e-mailed to dental practitioners, 286 e-mail replies were received from that e-mail list, indicating a response rate of 28.6%.

### Demographic and professional characteristics of respondents

About half of the questionnaire respondents were aged 20–34 years. More than half of the participants were general dental practitioners, males (56.3%), and one-third of them had less than five years of work experience. Over half of the practitioners worked in major cities (60%), and the remainder were working in smaller cities and towns in Saudi Arabia. Almost two-thirds of the respondents (67.1%) worked 35–49 hours per week. The majority of participants were working in public practices (51%), which was followed by private practices (32%) and academic practices (10%) (Table 1).

**Table 1:** Description of demographic and professional characteristics of participants.

Characteristics	Frequency	(%)
**Age (in years)**		
20–34 yr	136	47.6
35–44 yr	77	26.9
45–54 yr	57	19.9
55–64 yr	13	4.5
>65 yr	3	1.0
**Gender**		
Male	161	56.3
Female	125	43.7
**Qualification**		
Specialist	86	30.1
General dental practitioner	155	54.2
Resident/Graduate	6	2.1
Dental therapist	39	13.6
**Work experience (in years)**		
0–5 yr	96	33.6
6–10 yr	61	21.3
11–15 yr	44	15.4
> 16 yr	85	29.7
**Location of the main job**		
Major city	171	59.8
City/Town	107	37.4
Remote area	8	2.8
**Work setting of the main job**		
Private	90	31.5
Public	147	51.4
Both (private & public)	20	7.0
Academic	29	10.1
**Working hours per week**		
1–19 hr	30	10.5
20–34 hr	54	18.9
35–49 hr	192	67.1
50–64 hr	10	3.5
>65 hr	30	10.5

### Preferred methods of communication

**Fig 1:**
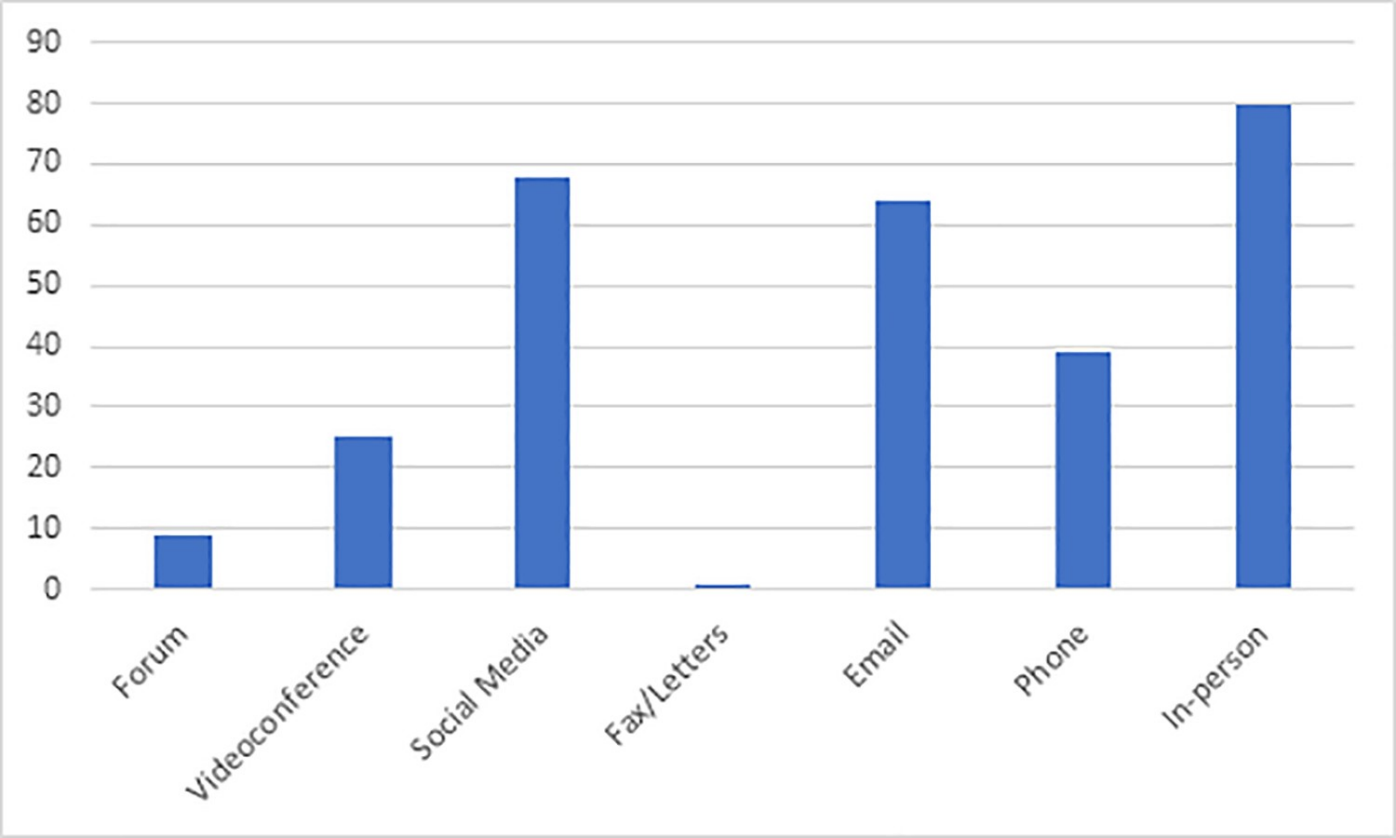
Preferred communication tools among dental practitioners.

There seems to be a shift in the paradigm of communication with the arrival of tele-technology, as traditionally used methods for communication, such as letters or fax, seem to be not preferable. The most popular method of communication chosen by the respondents were as follows: in-person (28%), social media (24%) and E-mail (22%). The least chosen communication methods among the respondents were the adoption of forums and video-conferencing. Fig 1 shows the response for the preferred methods of communications where subjects had the freedom to choose more than one option, therefore it was best to present the data in the form of a bar chart.

### Teledentistry confidentiality and security issues

When data are sent online, concerns about confidentiality tended to be higher than those about obtaining patient consent, hardware and software incompatibility, reliability of equipment, or digital forgery with just over 80% of respondents concerned about confidentiality. This was followed by potential digital forgery, where 77% of respondents expressed little or significant concerns. Concerns about incompatible hardware and software came in third place, where 76% of respondents expressed little or significant concerns. The reliability of teledental equipment was expressed by the respondents, where 41% were very concerned, and 34% were little concerned. The lowest levels of concern were seen by asking the respondents about gaining patient consent for teleconsultation, with just over 70% of respondents concerned about gaining patient consent (Table 2).

**Table 2:** Practitioners’ concern about data security and patient consent.

	VC (%)	LC (%)	NA (%)	NP (%)	NC (%)
Gaining patient consent for teleconsultation	116 (41)	89 (31)	39 (14)	21 (7)	21 (7)
Confidentiality when data are sent online	146 (51)	91 (32)	13 (5)	20 (7)	16 (6)
Potential for digital forgery	130 (45)	92 (32)	37 (13)	13 (5)	14 (5)
Incompatible hardware and software	95 (33)	122 (43)	43 (15)	15 (5)	11 (4)
Reliability of teledental equipment	117 (41)	96 (34)	39 (14)	20 (7)	14 (5)

VC = Very concerned, LC = Little concerned, NA = Not feeling either way, NP = Not particularly concerned, NC = Not concerned at all

### Teledentistry and practice improvement

Teledentistry was anticipated by most of the respondents to improve dental practice. More than 70% of respondents agreed that teledentistry would help reduce the waiting list, improve the interaction between colleagues, enhance advice and guidelines, provide a safe atmosphere for practicing dentistry, and make patient referrals more efficient. However, 40% of the respondents were unsure if teledentistry would help provide accurate diagnosis in a clinical setting (Table 3).

**Table 3:** Practitioners' perceptions of teledentistry's benefits in improving dental practice and patient care.

	DS (%)	D (%)	N (%)	A (%)	AS (%)
Practitioners' perception of the capability of the teledentistry to improve practice					
Teledentistry would provide accurate diagnosis in a clinical setting	16 (6)	73 (26)	108 (38)	70 (24)	19 (7)
Teledentistry would help shorten the waiting list	6 (2)	16 (6)	60 (21)	161 (56)	43 (15)
Teledentistry would enhance guidelines and advice	4 (1)	15 (5)	49 (17)	169 (59)	49 (17)
Teledentistry would improve the interaction between peers	10 (3)	6 (2)	65 (23)	154 (54)	51 (18)
Teledentistry would provide a safe atmosphere for practicing dentistry	8 (3)	11 (4)	42 (15)	127 (44)	98 (34)
Teledentistry would make patient’s referral more efficient	7 (2)	21 (7)	50 (17)	151 (53)	57 (20)
Practitioners' perception of the usefulness of the teledentistry for dental practice					
Teledentistry would enhance clinical training and continuing education	8 (3)	17 (6)	55 (19)	152 (53)	54 (19)
Teledentistry would reduce costs for the dental practices	11 (4)	27 (9)	93 (33)	130 (45)	25 (9)
Teledentistry would increase treatment time spent with the patient	12 (4)	93 (33)	74 (26)	98 (34)	9 (3)
Teledentistry would necessitate an extra appointment for taking photographs	6 (2)	33 (12)	93 (33)	136 (48)	18 (6)
Teledentistry would save time compared with a referral letter	5 (2)	11 (4)	73 (26)	164 (57)	33 (12)
Teledentistry would be too expensive to set up	24 (8)	76 (27)	96 (34)	81 (28)	9 (3)
Teledentistry would provide adequate diagnostic information	82 (29)	78 (27)	109 (38)	17 (6)	0 (0)
Practitioners' perception of the usefulness of the teledentistry for patients					
Teledentistry would save money for patients	4 (1)	24 (8)	79 (28)	154 (54)	25 (9)
Teledentistry would improve communication with patients	4 (1)	25 (9)	70 (24)	146 (51)	41 (14)
Teledentistry would be helpful patient education	4 (1)	7 (2)	47 (16)	146 (51)	82 (29)
Teledentistry would help to avoid unnecessary travel to Dental clinic	5 (2)	2 (1)	51 (18)	161 (56)	67 (23)
Teledentistry would be helpful in monitoring the patient's condition	5 (2)	10 (3)	59 (21)	154 (54)	58 (20)
Teledentistry would be convenient and well received by patients	3 (1)	22 (8)	90 (31)	145 (51)	26 (9)
Teledentistry would be useful for patients in remote areas	3 (1)	14 (5)	37 (13)	156 (55)	76 (27)
Teledentistry should be covered by dental insurance plans.	7 (2)	13 (5)	75 (26)	137 (48)	54 (19)

DS = Disagree strongly, D = Disagree, N = Neutral, A = Agree, AS = Agree strongly

### Teledentistry usefulness for dental practice

Teledentistry was found useful in improving dental practice by enhancing clinical training and continuing education and saving time compared to the conventional method of referral, according to a majority of the respondents. Over half of participants felt that teledentistry could be valuable in improving dental practice by reducing dental practice costs, but at the same time, they indicated that it would require extra time by arranging appointments for taking photographs. On the other hand, one-third of the respondents were less convinced that teledentistry would increase treatment time spent with the patient and be too expensive to set up. However, 40% of the participants were uncertain if teledentistry would help provide accurate diagnostic information (Table 3).

### Teledentistry usefulness for dental patients

About 60% of dental practitioners recognized the potential usefulness of teledentistry for patients. In addition, more than 70% of practitioners mentioned that teledentistry would be beneficial for patients residing in rural or remote locations. A similar agreement percentage was seen in regards to teledentistry's benefits in patient education and that it would help reduce the need to travel to the dental clinic as well it would be helpful to monitor the patient's condition. The rest of the questions covering the usefulness of the teledentistry for patients showed good response, where over 60% of practitioners agreed that teledentistry should be covered by dental insurance plans, would improve communication with patients, would save money for patients and would be convenient and well received by patients (Table 3).

**Fig 2:**
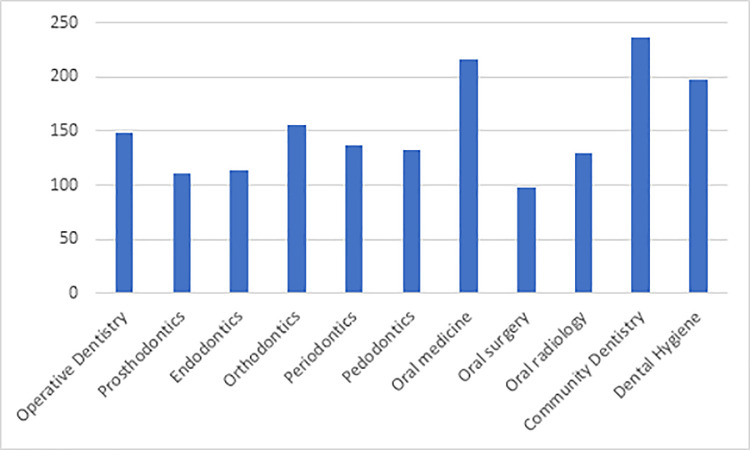
Preferred dental specialty for application of teledentistry.

The last question in the survey asked the participants about the preferred dental specialty for the application of teledentistry. Community dentistry came first, with over 80% of the response. This was followed by oral medicine (75%), dental hygiene (69%), and orthodontics with just over half of the response. The other dental specialties scored less than 50% of the response from the participants (Fig 2).

The ANOVA test shows the statistical importance of the participants' qualification as well as work experience in years with questions about teledentistry. For the qualification, the usefulness of teledentistry for patients was statistically significant (p = 0.027), where the general dentists scored lower than the others. As for work experience in years, both usefulness of teledentistry for the dental practice and patients were statistically significant (p = 0.046 and <0.0001 respectively). This could be explained by observing the mean scores between the groups, where those who worked for 11–15 years scored less than the other groups. On the other hand, there was no statistical significance between the participants' demographic variables, namely age and gender with questions about teledentistry as seen in Table 4.

**Table 4:** Statistical significance between the demographic variables of the respondents with the four domains of Teledentistry (ANOVA test).

Variable	Data security and patient consent Mean (SD)	The capability of teledentistry to improve practice Mean (SD)	The usefulness of teledentistry for dental practice Mean (SD)	The usefulness of teledentistry for patients Mean (SD)
**Age (in years)**				
20–34 yr	10.3 (4.0)	22.2 (3.6)	22.4 (3.6)	30.5 (4.5)
35–44 yr	9.0 (3.8)	22.0 (4.4)	22.1 (4.1)	30.5 (6.3)
45–54 yr	10.5 (4.9)	22.7 (4.0)	23.5 (2.9)	30.3 (3.8)
55–64 yr	8.7 (4.5)	23.6 (3.9)	22.8 (2.5)	32.5 (3.6)
>65 yr	7.7 (0.6)	18.3 (1.5)	19.7 (4.5)	28.0 (2.0)
**P-value**	0.098	0.215	0.122	0.567
**Gender**				
Male	9.6 (4.2)	22.2 (4.1)	22.5 (3.5)	9.6 (4.2)
Female	10.2 (4.2)	22.3 (3.6)	22.6 (3.7)	10.2 (4.2)
**P-value**	0.235	0.785	0.937	0.821
**Qualification**				
Specialist	9.2 (4.4)	23.1 (4.0)	22.9 (3.3)	31.5 (4.3)
General dental practitioner	10.0 (4.2)	21.7 (3.8)	22.2 (4.0)	29.8 (5.2)
Dental therapist	10.2 (3.9)	23.3 (4.8)	22.8 (3.3)	33.7 (3.8)
Resident/Graduate research	11.1 (3.7)	22.6 (3.5)	23.0 (2.7)	30.8 (4.5)
**P-value**	0.125	0.057	0.486	0.027*
**Work experience (in years)**				
0–5 yr	10.1 (3.9)	22.2 (3.4)	22.4 (3.6)	31.2 (4.5)
6–10 yr	10.0 (3.9)	22.3 (3.6)	22.1 (3.3)	31.0 (3.7)
11–15 yr	9.5 (4.2)	21.0 (5.1)	21.8 (4.7)	27.4 (7.1)
> 16 yr	9.8 (4.7)	23.0 (3.8)	23.4 (3.0)	31.1 (4.0)
**P-value**	0.853	0.050	0.046*	<0.0001*
**Range**	5–25	6–30	7–35	8–40
**Total**	9.9 (4.2)	22.3 (3.9)	22.5 (3.6)	30.5 (4.9)

*significant p-value

## Discussion

This study is the first to investigate Saudi Arabian dental practitioners' perceptions of teledentistry's usefulness in different aspects. Overall, a majority of the respondents expressed high agreement and positivity toward the use of tele-technology in dentistry. Most of the respondents (60%) felt that teledentistry would be beneficial to patients, especially those in remote locations, thus avoiding unnecessary travel to the dental clinic. They believed teledentistry could be useful in monitoring and educating patients. The overall results support the benefits of utilizing teledentistry for both dental practice and patients. Other published studies concerning teledentistry and dental practitioners' perception in other countries showed similar findings, where a majority of dentists stated the advantage gained by implementing teledentistry and how it benefited patients and the actual practice. [5, 12] Though the difference is not that high, it might be related to other concerns the study participants had regarding the patients' benefits.

In agreement with previous studies, there was considerable uncertainty about the possibility of teledentistry to help in providing accurate diagnosis compared to the clinical setting. [5, 7, 17–19] Other studies stated a promising diagnostic accuracy when diagnosing caries or oral pathology. [17, 20] This was achieved after proper training where training sessions were conducted for proper diagnosis by practicing dentists. Thus, teledentistry can produce acceptable diagnosis with proper training following a set protocol. Over half of the respondents indicated that teledentistry would necessitate an extra appointment to take photographs, this reflects the understanding of the need for proper photographs taken by trained personnel. [12] The highest concern (80%) reported was the concern about patient confidentiality when data are shared online, the ability to share health information via the internet is always a concern for patients and health professionals over who can view the information or have access to and this concern agrees with previously published literature. [18, 21, 22] However, the majority of the respondents agreed that teledentistry would be effective in reducing the waiting list, which is believed to be due to the effect of teledentistry on the referral process. Teledentistry can improve guidelines and peer communication; patients will be assigned to a dental specialty clinic based on an informed decision considering patients’ condition and needs. Patients management and referral process are some of the main drivers to impellent tele-technology in dental practice. [23–26]

Participants showed high agreement concerning the benefits the practice will gain when implementing teledentistry; they believed it would be advantageous in training and educating the practice personnel. Distance learning gave the dental personnel the chance to target the information needed at a time of their convenience, thus improving their skills and knowledge. [27–29] Respondents agreed with the statement that teledentistry could improve the practice by saving referral time and reducing the cost compared to conventional methods. Reduced cost and efficient time management can attract patients and gain their trust; published literature stated that tele-dental patient assessments were the lowest cost service model compared to conventional real-time consultation. [18, 30, 31] The majority of the respondents (<60%) agreed/agreed strongly that dental insurance plans should cover teledentistry cost, which can be another factor that attracts patients. Many countries have included teleconsultation and tele-practice into the health insurance plans. [32–34]

Participants showed high concerns relating to security, technical issues, digital forgery (76%), hardware incompatibility (78%), and equipment reliability (75%). To have the latest teledental technologies is not enough without the proper design of infrastructure. The solution must consider software/hardware compatibilities to ensure full secure integration between the different technical components. The right setup will reduce both the implementation and operation costs and errors. [10, 33–36]

It was expected that the age, work location, or working hours would influence the participants' opinion; in this study, those demographic factors did not affect the uses of teledentistry. Previous studies have shown that practitioners who work in remote areas or private clinics are more accepting of the tele-technology and its benefits. [11, 37–40] Other studies showed that those who work overtime would be keen to adopt tele-technology to reduce the working load. [41] The participants' qualifications and working experience were significantly affecting this study's results. In our study, general dentists scored lower than others; general dentists can demonstrate some resistance toward unfamiliar technology either due to lack of experience or misunderstanding the concept. Other published studies showed the excitement and the benefits gained by general dentists through tele-dental application; they were able to learn and sometimes perform more advanced procedures through tele-supervision. [11, 18, 42] Dentists with working experience of 11 to 15 years showed the lowest score, which is the middle age group, they are still in the process of navigating their way into building their career and might be resistant to the technology not to lose their patients pool.

When asked about which dental specialty can benefit from the application of teledentistry, the majority of respondents selected community dentistry followed by oral medicine, dental hygiene, and just over half-the participants selected orthodontics. This reflects narrower vision than expected; from the selection, it is obvious they limit their responses to screening, diagnosis, or patient education. Directed campaigns are needed to widen the perception and explain the full spectrum of teledentistry and its applications for dental professionals.

Earlier this year, most of the dental practitioners shifted their practice to teledentistry such as teleconsultation and tele-education in a way to cope with the COVID-19 pandemic.[43, 44] In order to gain control over the virus spread, routine dental care has been canceled during the pandemic, [45–47] teledentistry would be an excellent opportunity for the dental community to ensure continuity of care to patients despite the harsh circumstances with the pandemic.

The relatively high number of questions in the survey can be considered one of the limitations of this study; feedback from participants reported the survey's length as a complaint. In addition to the timing of conducting the survey within two months (March and April) which might explain the low response rate. Another limitation can be the use of the 5-point Likert scale; respondents tend to select the middle choices away from the edges rather than being sharply definite with their response. [48, 49] This survey reflects a positive perception and attitude toward teledentistry as a general concept, specific investigation targeting specific aspects such as the use of social-media in dentistry, patient screening, diagnosis, referral, or other implementation is needed. The results reflect the perspective of dental professionals, the readiness and willingness of the stakeholders in this case “the patients” is needed. The next rational step would be to investigate the knowledge and perception for specific selected applications of teledentistry then adopt a business model to implement the collected data and gather further information on the stakeholders’ perception, acceptance, and rising challenges to address.

## Conclusion

Generally, the feedback gained by this study showed readiness of the dental professionals to be engaged in the teledentistry approach. Despite the concerns reported, most participants agreed with the concept and showed a high understanding of the technical and ethical limitations when using teledentistry. With this study's limitation, further investigation is needed to understand the implementation and challenges of dental institutes and practitioners.

## Supporting information

S1 Appendix (DOCX)

S1 Data (SAV)
