# Supplementary material for: Teledentistry awareness among dental professionals in Saudi Arabia
Source: PLoS One. 2020 Oct 15;15(10):e0240825. doi: 10.1371/journal.pone.0240825 (PMC7561132; doi:10.1371/journal.pone.0240825)
Supplement: S1 Appendix — (DOCX) [file pone.0240825.s001.docx]

APPENDEX 1

**SURVEY**

**What is teledentistry?**

Teledentistry refers to the use of telehealth systems and methodologies in dentistry. Licensed or credentialed dentists and allied dental personnel are responsible for delivering services through teledentistry modalities.

Teledentistry can include patient care and education delivery using, but not limited to, the following modalities:

- **Live video:** Live, two-way interaction between a person (patient, caregiver, or provider) and a provider using audiovisual telecommunications technology.
- **Store-and-forward**: Transmission of recorded health information (for example, radiographs, photographs, video, digital impressions and photomicrographs of patients) through a secure electronic communications system to a practitioner, who uses the information to evaluate a patient’s condition or render a service outside of a real-time or live interaction.

**What prompts the need for teledentistry?**

The reason or reasons why a teledentistry event occurs depends on the circumstances, such as when all persons who must be involved are not able to be in the same physical location (Nowadays, the COVID-19 pandemic is one reason for the use of Teleldentistry).

**The following question will cover the demographic information of the participant:**

1. Age
   1. 20–34 yr
   2. 35–44 yr
   3. 45–54 yr
   4. 55–64 yr
   5. >65 yr
2. Gender
   1. Male
   2. Female
3. Qualification
   1. Consultant/Specialist
   2. General dental practitioner
   3. Resident/Graduate research
   4. Dental hygienist/therapist
   5. Other
4. Work experience (in years)
   1. 0–5 yr
   2. 6–10 yr
   3. 11–15 yr
   4. > 16 yr
5. Location of the main job
   1. Major city
   2. City/town
   3. Remote area
6. Work setting of the main job
   1. Private
   2. Governmental
   3. Both (private & governmental)
   4. Academic
7. Working hours per week
   1. 1–19 hr
   2. 20–34 hr
   3. 35–49 hr
   4. 50 + hr
8. Daily general purpose use of the internet (in hours)
9. <1 hr
10. 2–4 hr
11. 5–7 hr
12. 8–10 hr
13. >11 hr
14. Daily practice-related use of the internet (in hours)
    1. <1 hr
    2. 2–4 hr
    3. 5–7 hr
    4. 8–10 hr
    5. >11 hr
15. Preferred communication tool in your dental practice
    1. Forum
    2. Videoconference
    3. Social Media
    4. Fax/Letters
    5. Email
    6. Phone
    7. In-person

**Questions 11 to 15 will cover concerns about data security and patient consent.**

1. Gaining patient consent for teleconsultation
2. Confidentiality when data are sent online
3. Potential for digital forgery
4. Incompatible hardware and software
5. Reliability of teledental equipment

Choices:

1. Very concerned
2. Little concerned
3. Not feeling either way
4. Not particularly concerned
5. Not concerned at all

**Questions 16 to 21 will cover perceptions about the capability of teledentistry to improve practice**

1. Teledentistry would provide accurate diagnosis in a clinical setting
2. Teledentistry would help shorten the waiting list
3. Teledentistry would enhance guidelines and advice
4. Teledentistry would improve the interaction between peers
5. Teledentistry would provide a safe atmosphere for practicing dentistry (e.g., COVID-19 Pandemic)
6. Teledentistry would make patient’s referral more efficient

Choices:

1. Disagree strongly
2. Disagree
3. Neutral
4. Agree
5. Agree strongly

**Questions 22 to 28 will cover perceptions about the usefulness of teledentistry for dental practice**

1. Teledentistry would enhance clinical training and continuing education
2. Teledentistry would reduce costs for the dental practices
3. Teledentistry would increase treatment time spent with the patient
4. Teledentistry would necessitate an extra appointment for taking photographs
5. Teledentistry would save time compared with a referral letter
6. Teledentistry would be too expensive to set up
7. Teledentistry would provide adequate diagnostic information

Choices:

1. Disagree strongly
2. Disagree
3. Neutral
4. Agree
5. Agree strongly

**Questions 29 to 36 will cover perceptions about the usefulness of teledentistry for patients**

1. Teledentistry would save money for patients
2. Teledentistry would improve communication with patients
3. Teledentistry would be helpful patient education
4. Teledentistry would help to avoid unnecessary travel to Dental clinic
5. Teledentistry would be helpful in monitoring the patient's condition
6. Teledentistry would be convenient and well received by patients
7. Teledentistry would be useful for patients in remote areas
8. Teledentistry should be covered by dental insurance plans.

Choices:

1. Disagree strongly
2. Disagree
3. Neutral
4. Agree
5. Agree strongly

**Thank you for your kind and helpful participation. Last question below**

1. Teledentistry can be applied in which branch of dentistry? (choose one or more option)
   1. Operative dentistry
   2. Prosthodontics
   3. Endodontics
   4. Orthodontics
   5. Periodontics
   6. Pedodontics
   7. Oral medicine
   8. Oral surgery
   9. Oral radiology
   10. Community dentistry
   11. Dental hygiene
